# What complete mitochondrial genomes tell us about the evolutionary history of the black soldier fly, *Hermetia illucens*

**DOI:** 10.1186/s12862-022-02025-6

**Published:** 2022-06-01

**Authors:** J. Guilliet, G. Baudouin, N. Pollet, J. Filée

**Affiliations:** 1 EGCE, CNRS, 12 rue 128, 91190 Gif sur Yvette, France; 2 Cycle Farms, 6 boulevard de entrepreneurs, 49250 Beaufort En Anjou, France; 3 IDEEV - Laboratoire Evolution, Génomes Comportement, Ecologie CNRS Université Paris Sud UMR 9191, IRD UMR 247, 12 rue 128, 91198 Gif sur Yvette, France

**Keywords:** *Hermetia illucens*, Phylogeny, Mitochondrial genome, Phylogeography

## Abstract

**Background:**

The Black Soldier Fly (BSF) *Hermetia illucens* is a cosmopolitan fly massively used by industrial companies to reduce biowaste and produce protein and fat for poultry and aquaculture feed. However, the natural history and the genetic diversity of the BSF are poorly known. Here, we present a comprehensive phylogeny and time tree based on a large dataset of complete mitochondrial genomes better to understand the evolution and timing of the BSF.

**Results:**

In this study, we analyzed 677 CO1 sequences derived from samples found all over the five continents, leading us to discover 52 haplotypes, including ten major haplotypes. This worldwide cryptic genetic and genomic diversity is mirrored at a local scale in France, in which we found five major haplotypes sometimes in sympatry. Phylogenetic analyses of 60 complete mitochondrial genomes robustly resolved the phylogeny of the major BSF haplotypes. We estimate the separation events of the different haplotypes at more than 2 million years for the oldest branches characterizing the ancestral split between present North American lineages and the other highly diverse south-central American clades, possibly the following radiation beyond the isthmus of Panama northwards. Our data confirm that this North American lineage ultimately gave birth to almost all commercial BSF stocks that participated in the worldwide BSF dissemination through farm escapements.

**Conclusions:**

Our data resolve the phylogenetic relationships between the major lineages and give insights into the BSF’s short and long-term evolution. Our results indicate that commercial BSF stock’s genetic and genomic diversity is very low. These results call for a better understanding of the genomic diversity of the BSF to unravel possible specific adaptations of the different lineages for industrial needs and to initiate the selection process.

**Supplementary Information:**

The online version contains supplementary material available at 10.1186/s12862-022-02025-6.

## Background

The black soldier fly (BSF) *Hermetia illucens* (Linnaeus, 1758) is a Diptera of the Stratiomyidae family. The native area of the BSF is considered to be Central America and the northern part of South America, then spread to the entire continent over the last thousands of years [1, 2]. These geographical areas are now considered their natural area [1, 3]. The BSF is now a cosmopolitan fly found in tropical, subtropical, and tempered regions, from the 40th parallel north to the 45th parallel south [3, 4] where it is considered an exotic, non-invasive species, according to the current regulations [5–7]. This fly likely spread through shipping routes [8]. While studies based on naturalistic observations show that it was first discovered in Malta in 1926 [9], it has been suggested that the earliest record in Europe was in Italy about 500 years ago [10]. The BSF appeared in the early 1950s in the South-East of France [11] before spreading through the South-West [12] along the Rhône valley [13] and finally along the Atlantic coast [14]. Currently, the BSF can be found on the whole European territory, with few exceptions. It is reasonable to assume that the rise of international traffic and the industrial use of the BSF may have led to a steady flow of introductions in France and worldwide [15, 16].

The BSF was initially used in forensic entomology to date the post-mortem time [17]. Industrial companies heavily use it to reduce biowastes [18, 19], to produce protein [19–21], and fat [19, 20, 22]. These products are used to make animal feed [23] or biodiesel [24, 25].

BSF industries face similar issues as other sectors: knowing how to manage their species and genetic aspects to get the best out of it. To this end, it is crucial to know the genetic diversity of BSF existing internationally, nationally, and locally. Many articles exist about the nutritional aspect of the BSF [22, 26–29] or its microbiota [30–32], but few have focused on the genetic diversity of the BSF. We have some evidence of BSF genetic diversity from previous studies. Ten haplotypes based on the CO1 gene were found in South Korea with 245 individuals [33]. Still, on the CO1 gene and a worldwide sampling, 56 haplotypes were found with a divergence rate up to 4.9%, and it has been shown that individuals belonging to divergent lineages are still interfertile [34]. Finally, based on 15 microsatellite markers, 16 genetic clusters were found with hot spots in South America [2]. Other works focused on more fundamental aspects of BSF and assembled a reference mitochondrial genome and a chromosomal-scale nuclear genome [35–37].

Within the framework of the BSF industry, phylogeography can allow on the one side to account for genetic diversity at different scales (global, national, and local) and, on the other side, to have keys to the geographical origin of the species present in a territory or used by companies. This study may suggest local adaptations or some phenotypic plasticity due to habituation to different biomes.

Our study quantified genetic diversity at different scales: at the global level by studying the variation within the CO1 gene and more locally at the French level. Our report is based on data obtained by sequencing 114 new individuals for the CO1 gene focusing specifically on France (90 French individuals were analyzed) and extracting 563 CO1 sequences from databases. We confirm the hidden genetic diversity within *Hermetia illucens* at all scales of observation, allowing us to find nine different haplotypes. We sequenced and assembled 56 mitochondrial genomes from four different continents and 40 different locations. In addition, we collected sequencing data from databases allowing us to assemble three additional mitochondrial genomes. We also collected a mitochondrial genome already assembled from databases, resulting in 60 mitochondrial genomes. These data allowed us to resolve the phylogenetic relationships between the major haplotypes robustly. In addition, we also provide here the date of the separation events of the different haplotypes found. This separation turns out to be more than 2 million years old for the most distant haplotypes.

## Materials and methods

### Sampling

We decided to conduct a global sampling followed by a specific focus on France. A detailed table of samples and associated metadata is provided in (Additional file 1: Table S1). The origin of all CO1 sequences used in our study is summarised in Table 1, and a summary of sample numbers by continent is provided in Table 2.

**Table 1:** Origin of CO1 sequences and mitochondrial genomes

Origin	Number of individuals (CO1 sequences)	Number of individuals (mitochondrial genome)
Collection from the wild	91	43
Commercial origin	23	13
Mined from databases (GenBank and BOLD)	560	1
Assembled from SRA (raw sequences)	3	3
Total	677	60

**Table 2:** Geographic origin of CO1 sequences and mitochondrial genomes

Geographic origin	Number of individuals (CO1 sequences)	Number of individuals (mitochondrial genome)
South America	55	4
North America	43	2
Asia	277	4
Oceania	106	0
Africa	78	11
Europe (including France)	118 (90)	39 (38)
Total	677	60

Regarding the samples, one adult individual was found in Mexico in the wild in 2016. Individuals coming from Asia (Taiwan), Africa (Ghana and Kenya), and South America (French Guiana) were collected during the spring and summer of 2019. They come either from the wild or breeding farms (see Additional file 1). In France, the collection of BSF was done with the help of the citizen compost network [38] during the summer of 2020. We recovered mainly L5 larvae exclusively from shared and personal composts and some nearby adults. Between one and ten individuals were collected per area. In some French localities, collections in the wild have been made near BSF breeding areas (about 5 km). We obtained larvae in the areas of Blois, Bordeaux, Toulouse, Montpellier, Lyon, Paris. All these samples were stored in 100% ethanol at − 20 °C prior to DNA extraction.

In the NCBI Sequence Read Archive (SRA), we gathered all the CO1 sequences on NCBI and Bold and whole-genome sequencing data and reconstructed the mitochondrial genome. We were surprised in the Bayonne area to have BSF-like larvae, which turned out to be *Exaireta spinigera* larvae. These larvae were analyzed in the same way as the BSF.

### DNA extraction

We extracted DNA from larvae, pupae, and imago under the same conditions by grinding individuals in liquid nitrogen using a mortar and pestle. The ground samples were then processed according to the protocol of the Macherey–Nagel AXG 100 [39] kit for the isolation of genomic DNA from tissue with some modification: we added a second purification step with 3.5 ml of freshly prepared ethanol and centrifugation at 15,000 rpm at 4 °C for 15 min just before the final re-suspension. The DNA pellet was finally re-suspended in 100 μl of Tris 10 mM EDTA 1 mM pH 8.0. The purity was evaluated by spectrophotometry using a Nanodrop [40], and the concentration was quantified using the Qubit kit DNA Broad range [41]. DNA integrity was monitored using routine agarose gel electrophoresis.

### CO1 sequencing

We designed specific CO1 primers from the reference mitochondrial DNA [35] (Additional file 2: Table S2), then a mix was realized using NEB 5× standard buffer One Taq. The mix is then amplified in ONETAQ HOT 45 PCR. We purified the PCR products using the Illustra Exostar 1-step GE Healthcare kit before Sanger cycle sequencing. Cycle sequencing reactions were performed using the BigDye 3.1 chemistry, purified by precipitation, and sequenced using an ABI 3130 sequencer (Applied Biosystems). Base-calling was performed using the sequencing analysis software version 5.3 (Applied Biosystems). We assembled forward and reverse reads using the Geneious V R11 [42] assembler with the sensitivity set at medium/fast. The regions containing the primers were excised, and we kept only the sequences having more than 75% of high-quality base calls.

### Multiple sequence alignment and haplotype network reconstruction

We aligned CO1 sequences obtained in the laboratory and those recovered from the databases using the MAFFT [43] aligner included as a plug-in in the Geneious software [42]. We used the algorithm G-INS-i with a scoring matrix of 200 PAM/k = 2, a gap open penalty at 1.53, and an offset value at 0.123. We exported the multiple sequence alignment in nexus format [44] for haplotype network reconstruction and visualization using POPART [45]. We modified the output file to add geographical origins to the sequences.

### Shotgun sequencing and mitochondrial DNA assembly

A total of 56 BSF individuals were used for whole-genome shotgun sequencing using an Illumina method by Novogene UK [46–50]. The technique used is pair-end with a medium coverage rate of 4.68× (SD 0.50). The resulting sequences were checked for quality and counted for read parity. We take advantage of the fact that mtDNA is highly repeated in insect DNA. In insects, mtDNA represents on average 0.42% of the total DNA in the genome sequence project [51]. As stipulated, we used 5000 kb of data/sample (4–5× coverage) for the mitochondrial assembly, which on average contains (5000 * 0.42)/100 = 21,000 kb of mtDNA. As the mitochondrial genome is about 15 kb, we obtain coverage of 1400× for the mitochondrial genome, which is mainly sufficient to obtain a complete genome in one contig. The sequences were assembled de novo with the MEGAhit software [52] (based options); the mitochondrial genomes were thus recovered from the assemblies by blast [53] using the reference BSF mitochondrial genome sequence (NC_035232). The mitochondrial DNAs were annotated using Mitos online software [54], and the D-Loop zone was removed. We added four complete mitochondrial sequences from databases or reconstructed them via reading sequences available on the SRA [55] using the same assembly procedure and then aligned them under the same conditions.

### CO1 and whole mitochondrial genome phylogeny

We use the MEGAX [56] software to determine the best model and compute an ML Tree with 500 Bootstraps using a complete deletion parameter. We visualized the data with the online tool ITOL [57], and we rooted the tree with the Stratiomyidae *Exaireta spinigera*. We chose *Exaireta spinigera* because it was the closest species for which we have a complete mitochondrial genome (the blastn result of the complete mitochondrial genome of *Hermetia illucens* vs. *Exaireta spinigera* gives us 81.28% of similarity). To simplify the interpretation of the phylogeny, we have kept only a single identical sequence per haplotype.

### Time tree

We used the BEAST software [58] to estimate the divergence time of the different BSF groups. The analysis was performed with an expansion growth model and an uncorrelated lognormal relaxed clock with the proposed insect molecular clock. Ten million iterations were performed, and then with the TreeAnotator [59], the obtained trees were clustered with a burn-in of 25% of the trees. The visualization of the final tree was done with the online software ITOL [57].

## Results

### Haplotype network

**Fig. 1:**
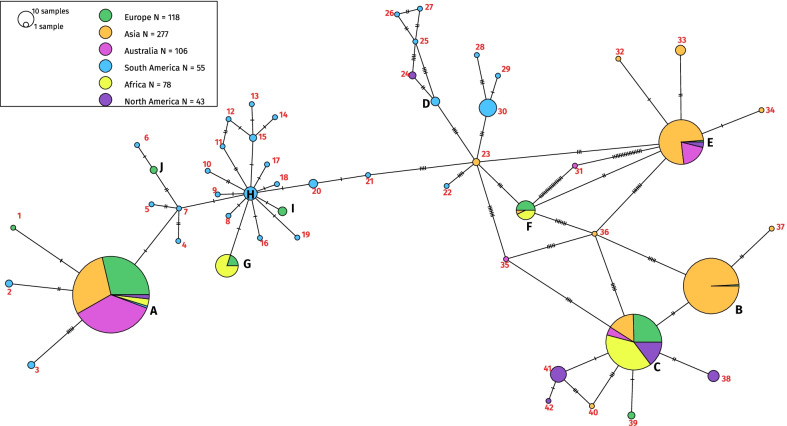
CO1 haplotype network of *Hermetia illucens*. The haplotype network generated by PopArt after multiple alignments are based on the CO1 gene of *Hermetia illucens*. Analysis of the differences between the CO1 gene sequences of 677 individuals (we did not separate commercial and wild individuals). The pie charts represent the same haplotype, and their relative size indicates the number of individuals sharing this haplotype. The number of bars between each segment indicates the differences between the two sequences. Within a pie chart, the colours represent the geographical origin of the individuals. The major haplotypes include more than five sequences (A, B, C, E, F, G, H, 30, 38 & 41). We grouped individuals by continent, separating North America from Central and South America. The list of sequences and their exact origin is available as an Additional file 1

We reconstructed a haplotype network from the analysis of 677 sequences spanning 658 nucleotides of the CO1 mitochondrial gene. Using the minimum spanning method [60], 52 distinct haplotypes were delineated from the analysis of this CO1 fragment. These different haplotypes group up to 230 sequences (Fig. 1).

There are 31 haplotypes containing only one sequence and 11 haplotypes with between 2 and 4 sequences from the same continent. We have named these 42 haplotypes “minor haplotypes.” We observed ten haplotypes between five and 230 sequences, including six multi-continental haplotypes that we will refer to as major haplotypes. We found an important diversity both within and between continents using the CO1 phylogenetic mitochondrial gene. Minor haplotypes represent 8.5% of all sequences (58 sequences) and 80.8% of haplotypes. Major haplotypes represent 91.5% of the sequences (619 sequences) and 19.2% of the haplotypes.

In Europe, we found ten distinct haplotypes from 118 sequences, nine for Asia from 277 sequences, five for Oceania from 106 sequences, 30 for South America from 55 sequences, four in Africa from 78 sequences, and seven in North America from 43 sequences. Some major haplotypes are represented in a more restricted area. For example, haplotype B is mainly in Asia (Fig. 1). We observed a high haplotype diversity in South America with 28 minor haplotypes that contain between one and four sequences. The haplotype C (we choose C for “commercial”) contains all the sequences derived from farmed strains of the industry working on BSF.

### Global distribution analysis

**Fig. 2:**
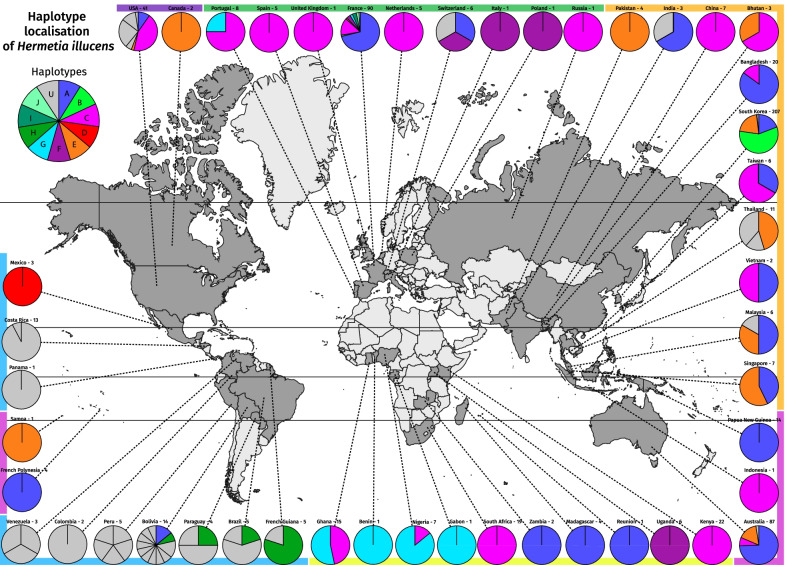
World CO1 haplotype distribution. Global distribution of the different *Hermetia illucens* haplotypes based on the CO1 gene. The pie charts represent the proportion of each haplotype within a country. The country’s name is followed by the number of individuals analysed. A coloured band representing the continents is next to each pie chart. The colours represent the haplotypes determined in Fig. 1. The haplotypes represented with grey colours, indicated with the letter U, are represented with a number (1–42) in Fig. 1. Separation is made in the pie chart when the haplotypes change for the same country. For example, Venezuela contains three unique haplotypes, meaning that these three individuals are different. A summary table can be found in Additional file 1

We visualized the actual distribution of the different haplotypes found previously on a distribution map (Fig. 2).

Each haplotype from A to J is now represented with a color (Fig. 2); haplotypes numbered in the haplotype network (Fig. 1) are now represented in grey.

We observed great variability in South America, highlighted on the map by the grey pie charts representing mostly minor haplotypes. We observe 22 singletons in South America out of the 52 haplotypes found, more than 40% of the total diversity. On the other hand, we found six out of ten major haplotypes in Europe.

Haplotype A and C were the most represented haplotypes in our sampling: Haplotype A was present on all continents and represented 34% of all sequences, and haplotype C was only absent from South America and represented 18% of all sequences. Some haplotypes seemed to be restricted to some world areas, such as haplotype G, which was present in all the localities in West Africa and surprisingly in a Portuguese sample, or the haplotype H in South America. Haplotype F was present in Europe, Uganda, and South Korea. Haplotype E was more present in Asia, Oceania, and North America. Finally, some haplotypes such as B, I, and J present rare sequences found in more restricted areas such as B in Korea or H and J in France (Fig. 2).

### CO1 phylogeny

During our CO1 gene analyses, some BSF-like larvae were identified as *Exaireta spinigera* larvae. The sequences were amplified and sequenced with the same primers as for BSF.

**Fig. 3:**
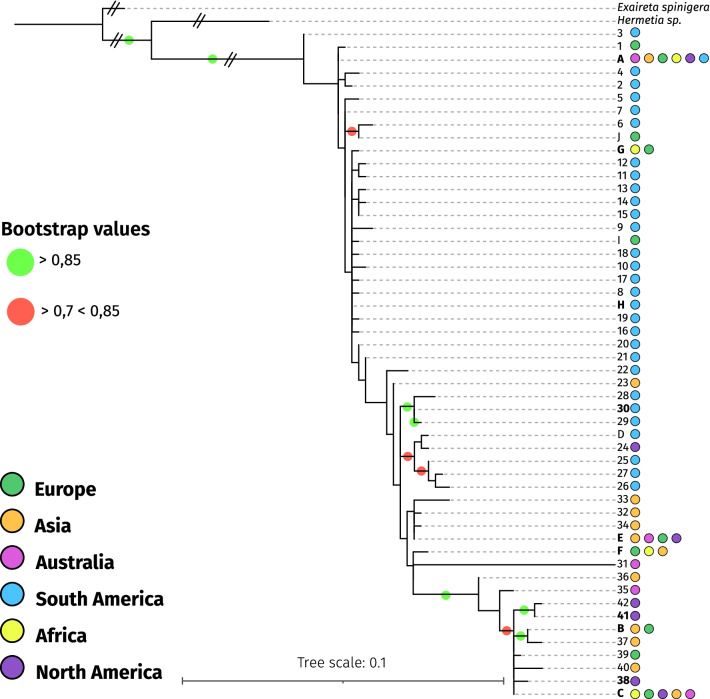
CO1-based phylogenetic tree. ML phylogenetic tree. Rooted phylogeny with *Exaireta spinigera* (Stratiomyidae). Bootstrap values are indicated with coloured circles: green for values > 0.85 and red for values between 0.7 and 0.85. We only kept one sequence per haplotype to visualise the relationship between them. Next to each haplotype, coloured circles represent the geographical origin of the species present in this haplotype. The order indicates the relative abundance of the individuals in each haplotype. The statistical support was estimated with 500 Bootstraps with MegaX software using the Tamura 92 G+I model

By taking only one sequence for each haplotype, we have reconstructed a phylogeny of the CO1 gene sequence. The CO1 phylogeny was rooted with the *Exaireta spinigera* (Fig. 3). A sample from Cameroon was revealed to belong to a closely unidentified species of BSF and was termed *Hermetia* sp. Cameroon in our subsequent analysis (Blastn score between *Hermetia* sp. from Cameroon and BSF gives us 85.01% identity with an E Value of 2e−174).

The CO1 gene phylogeny supports the analyses performed with the haplotype network (Fig. 1), showing remarkable sequence diversity among the CO1 gene. Interestingly, all the commercial strains are found in the C haplotype (a detailed phylogenetic tree is given as a supplementary with an asterisk placed on individuals from farms (Additional file 3: Fig. S3).

A group containing haplotypes 35 to 42 and haplotypes B and C forms a divergent group from the others, with strong statistical support (bootstrap value of 0.99). Some individuals captured in the wild have been found near BSF commercial breeding sites. For example, we have captured C haplotype individuals in Avignon close by companies working with the BSF.

Although the global resolution of the deep branches of the tree is low, it is also possible to better understand the relationship between the haplotypes: haplotypes B and C are close to each other and well separated from other haplotypes. The other haplotypes are not very well resolved with the CO1 sequences. Finally, the samples coming from Latin America are scattered all over the tree, showing their great diversity and supporting the hypothesis of the geographical origin of the BSF.

### France displays vast BSF genetic diversity at local scales

**Fig. 4:**
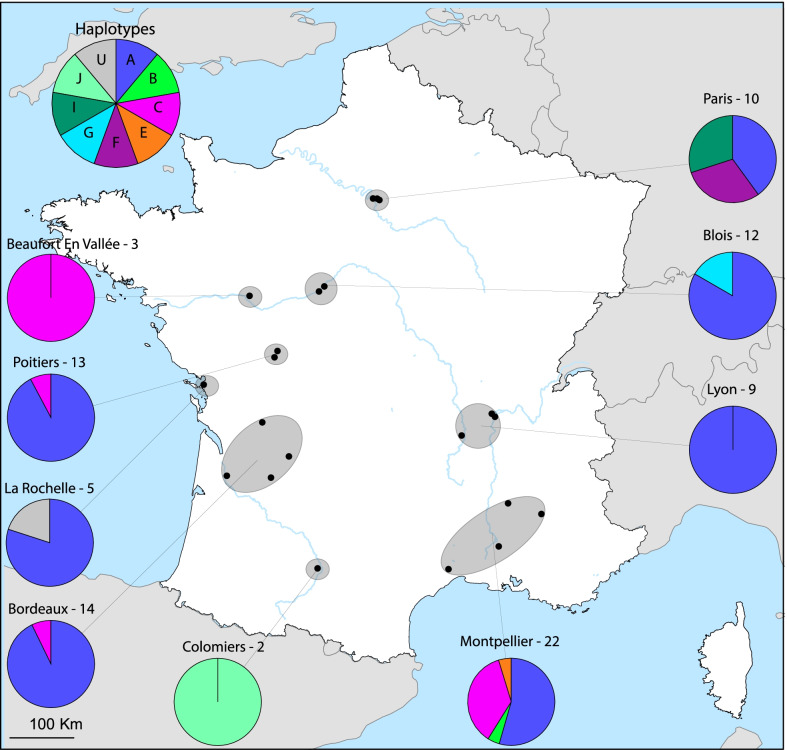
France CO1 haplotype distribution. Distribution of the different haplotypes of *Hermetia illucens* in France. The dots represent the specific area of collection. The pie charts represent the distribution of haplotypes in the corresponding area. The names of the main cities in each area are indicated above, and the number of sequenced individuals. Paris includes Vincennes and Nogent Sur Marnes. Blois includes Valloire Sur Cisse. Lyon includes Saint Marcellin En Forez, Corbas, Champagne Au Mont d’Or and Dardilly. Montpellier includes Avignon, Trescléoux and Dieulefit. Bordeaux includes Lormont La Force and Périgueux. Poitiers includes Saint Georges Lès Baillargeaux

Among the ten major haplotypes, our sampling effort in France showed that five are present in France, and a unique haplotype is only found in France (Fig. 4) in La Rochelle. Haplotype A remains the dominant haplotype in France (71.1% of all sequences) in seven of the eight localities (excluding Beaufort en Vallée, where the flies come from a company). Regarding the haplotype C, BSF belonging to this group have been found in four locations in the regions of Poitiers, Bordeaux, Beaufort-En-Vallée and Montpellier. Except for Beaufort-En-Vallée, where the flies come from a company, the other BSF come from natural environments. BSF industries exist in all these localities. More surprisingly, different haplotypes coexist in some localities. For example, we found three different haplotypes in the region of Paris and four in the region of Montpellier. Finally, we found a singular haplotype in Colomiers (J) and La Rochelle (1).

### Complete mitochondrial genome analyses

**Fig. 5:**
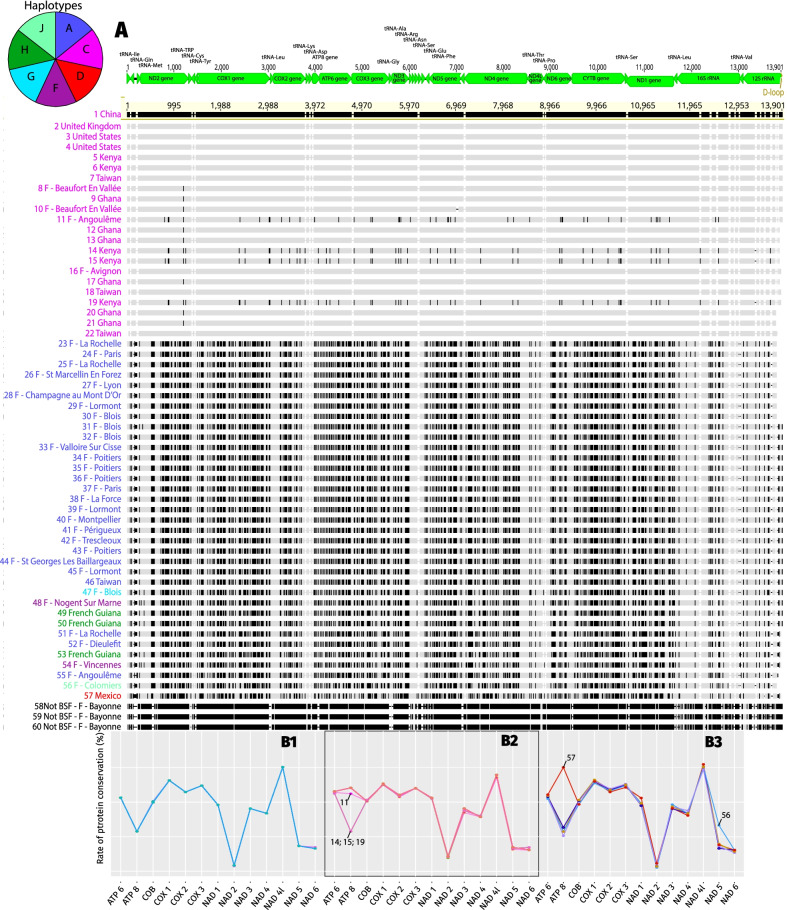
Mitochondrial DNA alignment and sequence conservation between *Hermetia illucens* and *Exaireta spinigera*. **A** Multiple alignments of mitochondrial DNA sequences from *Hermetia illucens*. Alignment was performed with the MAFFT aligner. The colours of the names come from their haplotypes determined with the CO1 sequences. Each black mark represents a difference with the reference (1—China). Above the alignment, there is a linear representation of the mitochondrial genes (a circular one can be seen in Additional file 4). The three last sequences are those of *Exaireta spinigera* analysed in the same way as *Hermetia illucens* (we kept one that we used as an outgroup because it was the closest complete mitochondrial sequence phylogenetically that we had). The sequences are numbered to be found in Fig. 6. **B** Sequence conservation of mitochondrial genes of *Hermetia illucens* to *Exaireta spinigera*. The sequences are grouped according to haplotypes. The A haplotype (**B1**), the C haplotype (**B2**), and the other haplotypes (**B3**). The sequences inside each graph are represented in the alignment (5A). Sequences from 23 to 46 plus sequences 51, 52, and 55 for haplotype A (**B1**); from 1 to 22 for haplotype C (**B2**); and from 47 to 57 (except sequences 51, 52, and 55) for the other haplotypes (**B3**)

To better understand BSF phylogeny and estimate the timing of haplotype divergence, we analyzed the mitochondrial genomes of 60 individuals (56 that have been sequenced and assembled, three from which sequences have been recovered and assembled and one recovered), including 3 *Exaireta spinigera* genomes (used as an outgroup). The 60 reconstructed mitochondrial genomes have the typical structure of insect mtDNA: 13 protein-coding genes, 22 tRNA, two rRNA-coding genes, and non-coding regions and control regions (an example is given as a Additional file 4: Fig. S4). The genetic differences are homogeneously distributed overall mtDNA and are not localized on specific genes (Fig. 5A). A great genetic diversity at the mitochondrial genome level is observed. Two very homogeneous groups, A and C, are well represented, and sequences of the C haplotype display many mutations all along the genomes from the others. The two sequences with the most differences are 55-F-Angouleme and 21-Ghana, with a similarity percentage of 96.373% (all results are in Additional file 5. Shorter alignment lengths result from comparisons with the outgroup sequences that split the alignments into two fragments.) The mitochondrial genomes of the three *Exaireta spinigera* were extremely close to each other (100% by BLAST—Additional file 5), so we kept only one to serve as an outgroup.

We also wanted to know if the mutations are equally (or not) distributed between the different genes. Therefore, we compared the similarity of the 13 mitochondrial genes of the 57 BSFs (Fig. 5B) to those of *Exaireta spinigera*. The results are presented as percent sequence similarity.

We grouped the individuals into three different categories:

Haplotype A (Fig. 5B1) represents the percentages of differences of the 27 individuals of haplotype A compared with *Exaireta spinigera*. All individuals show the same level of gene difference as those of *Exaireta spinigera*. Only the NAD6 gene presents slight differences.

Haplotype C (Fig. 5B2) represents the percentages of differences of the 22 individuals of haplotype C compared to *Exaireta spinigera*. It shows that most of the mitochondrial genes are identical except for the ATP8 gene, which is different for four individuals (11 Angouleme; 14 Kenya; 19 Kenya, and 15 Kenya). Slight differences are also present in the NAD3 and NAD6 genes.

Haplotype D–F–G–H–I (Fig. 5B3) represents the percentages of differences of the eight other individuals of haplotype C compared with *Exaireta spinigera*. The profiles are slightly different, with a more substantial disparity for the ATP8, NAD1, and NAD5 genes, especially the Mexican one (57). The profiles show the same pattern except for the ATP8 gene of the individual from Mexico (57), which is more conserved.

### Complete mitochondrial genome phylogeny

The complete mitochondrial genome phylogeny demonstrates the very high genetic divergence among BSF populations found with the CO1 gene analysis (Additional file 7: Fig. S7). The increased sensitivity of the analysis, due to the size of the sequences (13.96 kb, 13 genes), robustly resolves the relationships between different haplotypes identified previously (nodes > 95%). In addition, it allows us to distinguish subgroups within the previously determined haplotypes. Two major divisions emerge within the tree: group C (which includes all commercial strains) and others. Two subgroups emerge within the C group (11—F—Angouleme; 14 Kenya; 19 Kenya; 15 Kenya and the others).

The other large group contains sequences of A, D, I, G, and H haplotypes. The I G and H haplotypes are related and close to each other. The very cosmopolitan A haplotype is well conserved with limited mitochondrial sequence divergences.

Moreover, although phylogenetically close, the I, G, and H haplotypes are found in very disparate areas (the G haplotype is mainly in Africa, the I in Europe, and the H in Latin America), supporting multiple worldwide introductions.

Strikingly the high level of the conservation of the ATP8 sequence described above in the Mexican sample (haplotype D) does not explain the basal position of the sequence in the phylogeny; indeed, we tested the phylogeny by removing the ATP8 gene, and no change was found (Additional file 6: Fig. S6).

The ATP8 gene alone is not sufficient to explain the appearance of the observed subgroups. However, unlike the CO1 gene, which varies only slightly within this haplotype, the NAD2, NAD3, NAD5, and NAD6 genes are more variable.

The average percentage of identity between haplotypes A and C is about 96.39%. Between haplotypes C and D, 96.54%. Between haplotype C and H, 96.40% and 96.43% between C and I. Between A and D, 97.82% (all results are in Additional file 5: Fig. S5).

### The divergence time of the different groups

**Fig. 6:**
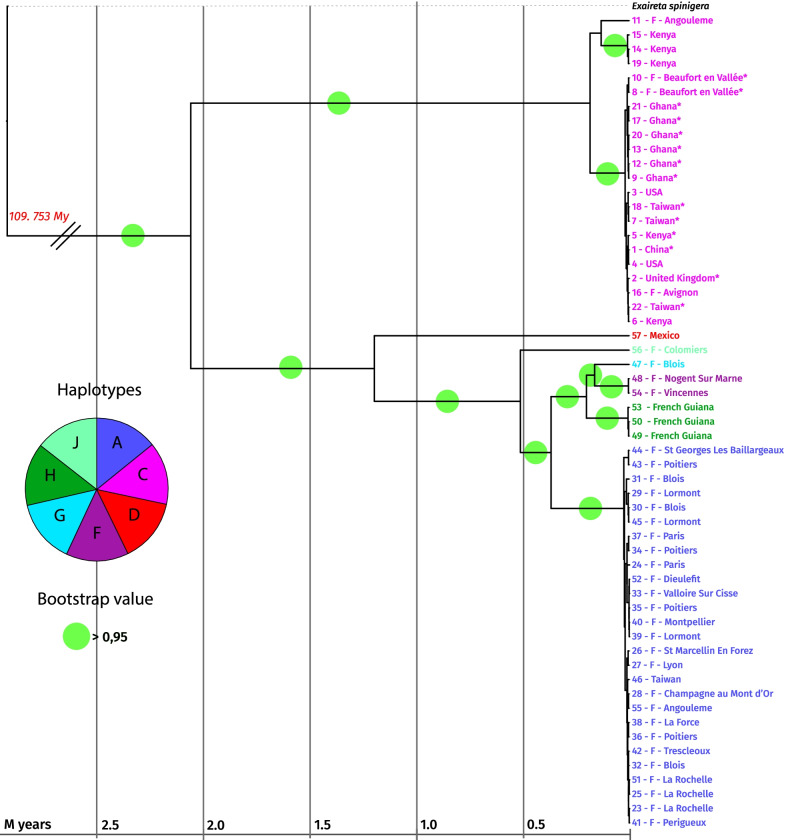
Mitochondrial DNA phylogenetic and time tree. Bayesian time tree obtained with BEAST of 57 mitochondrial genomes of *Hermetia illucens* deprived of D-Loop regions, aligned with MAFFT and rooted with *Exaireta spinigera*. The scale is in millions of years. A break in scale is made at the root level. The colours of the names correspond to the haplotypes determined with the CO1 sequences (Fig. 1). Commercial individuals are marked with an asterisk. The green circles come from the ML phylogenetic tree of the 57 mitochondrial DNA sequences of *Hermetia illucens*, deprived of D-Loop regions. MAFFT was used to align the sequences, 500 Bootstrap replicas were performed. The outgroup used is *Exaireta spinigera* (Stratiomyidae). Bootstrap values are indicated by a green circle when they are > 0.95 (the separate trees can be seen in Additional file 7)

The time tree (Fig. 6) gives us information about the divergence time of the different mitochondrial genomes. The phylogenetic tree presented in Fig. 6 contains both the Bayesian and the ML trees. The two separate trees are presented in Additional file 7. We noted a relatively long divergence time between haplotype C and the others (2.2 million years). The others have diverged more recently (about 1.2 million years). Within the C haplotype, a divergence has been about 300,000 years, while in the other groups, the divergence times are much lower, about 20,000 years for the A haplotype.

The time of divergence with *Exaireta spinigera* has been estimated at 110 million years, confirmed by the literature that gives us 112 million years with the Timetree tool [61].

## Discussion

*Hermetia illucens* is now considered a species of major interest for the feed industry [62, 63]. Thus, it became urgent to study the genetic diversity within the different populations at different scales. Analyzing the different haplotypes in the same geographical area is a powerful tool for studying the recent history of BSF. Hence, an in-depth study of the genetic variations between the different haplotypes may allow us to resolve better the scenario of the arrival of BSF in a territory.

Our study uncovered a high genetic variability of mitogenomes within the species *Hermetia illucens*. We found that haplotype C is the most divergent. The dating of the division between the C haplotype and the others goes back more than 2 million years. We found a well-resolved separation within haplotype C (> 95%); the other haplotypes are also well resolved (> 95%).

Our study confirms the genetic diversity found in previous papers [2, 33, 34]. We found a comparable number of haplotypes, although we added many more sequences of diverse origin but mainly from non-indigenous areas. For example, Ståhls et al. [34] found 56 distinct haplotypes with 418 CO1 sequences, and we found a comparable number of haplotypes based on the analysis of 677 CO1 sequences. These results suggest we have now captured a major part of the extant BSF worldwide diversity outside of the genetic hotspots identified previously [2, 34], namely Latin America. Indeed, in South America, we found 30 haplotypes among the 55 sequences sampled that open up the possibility of a still unknown genetic diversity in this part of the world.

With the maximum diversity found in Latin America, the hypothesis of the geographical origin of BSF [1] is supported by our study. Analysis of the complete mitochondrial genome supports the hypothesis of several distinct introductions on all non-native continents. Considering the estimated divergence times between the different haplotypes, it seems impossible that this is due to a single introduction followed by diversification in the territory.

On the other hand, according to Kaya et al. [2], an alternative but complementary scenario is also possible: bridgehead populations, consisting of a mixture of haplotypes, have been introduced on different continents. Indeed, taking Europe as an example, the eight haplotypes found in Europe can be explained by the admixture scenario proposed by Kaya et al. [2], who argue that central European BSF populations originated from admixture between Australian and Southeast African populations, which themselves can be traced back to earlier independent admixture events. Furthermore, Kaya et al. [2] suggested some gene flows with Mediterranean (altogether originating from Latin America) and particularly East African populations, which interestingly seems to be corroborated in our study, as exemplified by the F haplotype. It would be extremely useful in the future to combine nuclear data and mitochondrial data to better resolve the potentially diverging patterns of the different markers and to precisely estimate relative impacts of historic genetic admixtures versus recent introductions on regional population structures. France contains on its territory a great diversity of haplotypes, whose origin can be explained either by the historical introduction around the year 1950 [11] in addition to more recent and repetitive introductions. The haplotype A, which is by far the most common haplotype in wild BSF found in France (71.1%) and in Europe, may correspond to the initial introduction. On the other hand, our data suggest that the presence of haplotype C in France may be due to a more recent introduction caused by BSF industries.

As suggested in a recent article on the structure and demography of the BSF based on microsatellite data [2], the captive populations used for breeding in Europe and North America derive from a common, genetically related strain. Our analysis based on CO1 and complete mitochondrial genome sequences confirm this result: the haplotype we named C contains all the sequences of industrial origin with very little genetic diversity. Interestingly, this commercial group does not have any South American relatives. Based on nuclear genetic data, Kaya et al. report wild North American populations being most closely related to the most prevalent managed strains, which we infer to be dominated by haplotype C. This result reinforces the hypothesis of a North American origin of the BSF commercial stock.

Moreover, specific individuals in the wild belonging to this group were collected in the vicinity (around 5 km) of industries breeding BSF. For example, if we focus on the commercial haplotype analysis in France, the wild BSF belonging to haplotype C may have escaped from industries in Poitiers, Bordeaux, Avignon, and Montpellier. This last point raises the question of the biosecurity of BSF farms worldwide. We can assume that the priority of companies involved in BSF farming is not biosecurity and escape control. Indeed, costs in terms of security to prevent insects from escaping are high, and the regulation is late at this level. In that case, the continuous flow of escaping BSF of industrial origin in large numbers could either result in an establishment in territories where it was not yet present or trigger losses of genetic integrity of locally resident wild populations via continuous one-directional introgression. This would lead inexorably to a loss of the local genetic diversity that may prevent future adaptations to changing environmental conditions.

However, it is interesting to note that in Ståhls et al. [34], in contrast to our study, some genetic diversity in the breeding strains in Europe and North America has been evidenced (10 haplotypes). This may be due to a broader sampling effort of commercial strains. Indeed, Stahls [34] had the opportunity to sample many small farms leading to 292 BSF sequences from rearing cultures. This allowed them to access a broader genetic diversity not found in our study. It is also possible that smaller farms did not obtain BSF from the dealers who constituted most of the more prominent industries but directly from the wild. Nevertheless, our results confirm that the industrial actors work with the same and unique haplotype.

We also evidence that the mitochondrial genome is ideally suited to resolve long-term BSF phylogenetic relationships. It allows us to date the natural history of the BSF, bringing a sum of details that were impossible to distinguish based only on the CO1. For example, we report puzzling variations in the level of conservation of the ATP8 genes for some samples (haplotypes C and D). Interestingly, these variations have already been found in other insect species (for example, in Coleoptera, see Zhang et al. [63], and in grasshoppers, see Li et al. [64]). The ATP8 gene is small (160 bp on average in insects) and encodes for a subunit of the complex V of the ATP synthase. In *Hermetia illucens*, the initiator codon of the ATP8 gene is ATT, is 167 bp long, and it overlaps with the ATP6 gene. It has been assumed in other species that the modification of the sequence of the ATP8 gene can affect the structure of mitochondrial complex V and thus its function.

Furthermore, it has been shown that changes within ATP synthase can be explained by adaptation to different ecological environments [65–67]. This indicates that some local adaptation driven by the ATP8 genes may exist in BSF, as it has already been shown in other insects. This can lead to an adaptation to survive in high-altitude environments, i.e., at a lower oxygen level and low temperature [68]. Even if these variations did not change the phylogeny of the BSF, it could be a clue to some local adaptation signal. The fact that all commercial strains (plus USA plus Mexico) show a high level of conservation of the ATP8 gene compared to all other wild strains may suggest some adaptation to rearing.

Complete mitochondrial genome data allows us to make a robust and well-resolved time tree of the species. The divergence times between the different haplotypes are surprisingly long (more than 2 million years for the most divergent lineages), and the differences observed between the sequences prove that there is still unsuspected and hidden genomic diversity. Comparing it to *Drosophila*, 2 million years correspond to many speciation events leading to different reproductively isolated species [69]. By contrast, Stahls et al. [34] indicated that BSF belonging to phylogenetically divergent haplotypes can still cross and generate offspring, indicating no apparent reproductive isolation in laboratory conditions. Interestingly, our data evidence that the cohabitation of different haplotypes at the same locality could lead to crosses between haplotypes. According to our results, the 2 million years of divergence between the C haplotypes and the other haplotypes could correspond to the North American lineages separating from the ancestral BSF population in South America upon their northwards range expansion beyond the Isthmus of Panama, which appears 2.8 million years ago [70]. This seems especially true since the C haplotype has not been found in South America but only in North America. This ancestral and natural colonization of North America more than 2 million years ago may also explain why the C haplotype group displays a higher degree of mitochondrial genetic diversity than the other groups, a process already observed in other insects as ants for example [71]. Further investigations are thus needed to understand the possible genetic barriers between highly divergent BSF groups but also the possible patterns of introgression in natural BSF populations. This phenomenon might considerably confuse the elucidation of the population structure and the natural history of these species.

Finally, it is interesting to recall that, if indeed, most industrial actors use the same haplotype for the industry, the other haplotypes present in France are highly divergent from this one. Strikingly, we found in one compost bin in the region of Paris flies belonging to two different haplotypes (A and F). Thus, crossbreeding between these two individuals belonging to these two haplotypes would bring a solid genetic mix that could benefit breeding. Associated with this significant genetic diversity, BSF may also have some significant phenotypic diversity that might be useful for specific industrial usage and selection processes to increase performances or ameliorate peculiar traits or behaviors [72].

## Conclusion

Through the analysis of 60 mitochondrial genomes during this study, we proposed a robust and well-resolved time tree of the BSF that confirms the vast genetic diversity within *Hermetia illucens*. Our result elucidates the phylogenetic relationship and time divergence between the BSF lineages. By adding almost 20% of CO1 sequences, mainly from non-native areas, we did not significantly increase the number of haplotypes previously found [34]. This indicates that a significant part of the diversity from non-native areas has already been found and that we should now concentrate our efforts on native areas. Furthermore, the combination of our results with the nuclear analysis already performed [2] gives an interesting insight into the invasion routes of the BSF and the divergence times between the different groups. We have also shown that many BSF industrial companies work with flies derived from a unique haplotype. We would also like to recall that the apparent biosecurity deficiency within some BSF industrial companies probably led to a continuous flow of escapement and possible local introduction/replacement of natural populations of BSF. Finally, our data indicated that it becomes fascinating to look at the nuclear genomes’ evolution of the BSF to better understand the specific adaptations of the different lineages that might be useful for industrial needs and initiate the selection process for peculiar traits.

## Supplementary Information

**Additional file 1.** List of all individuals used during the study. Origin and accession number.

**Additional file 2.** CO1 gene sequencing protocol. Primers used and Tm.

**Additional file 3.** Phylogenetic tree of the CO1 gene.

**Additional file 4.** Annotated mitochondrial genome of Hermetia illucens.

**Additional file 5.** Paired blasts of the mitochondrial genomes used in the study.

**Additional file 6.** Phylogeny based on the complete mitochondrial genome of Hermetia illucens with the ATP8 gene sequence removed.

**Additional file 7.** Mitochondrial DNA phylogenetic tree and mitochondrial DNA time tree.

## Data Availability

The dataset generated during the study has been deposited in the European Nucleotide Archive database under Accession Numbers PRJEB48031 Bioprojet. Alignment and raw data can be downloaded following this link: https://figshare.com/articles/dataset/sequences_and_trees_zip/19589335. The supplementary can be downloaded following this link: https://figshare.com/articles/dataset/Supplementary_zip/19589332.
