# Supplementary material for: What complete mitochondrial genomes tell us about the evolutionary history of the black soldier fly, Hermetia illucens
Source: BMC Ecol Evol. 2022 Jun 1;22:72. doi: 10.1186/s12862-022-02025-6 (PMC9158166; doi:10.1186/s12862-022-02025-6)
Supplement: Supplementary file 2 — Additional file 2. CO1 gene sequencing protocol. Primers used and Tm. [file 12862_2022_2025_MOESM2_ESM.pdf]

CO1 gene sequencing protocol. Primers used and Tm.

ADN (10 – 50 ng)	2µl
H20 (qsp 25µl)	12,37 µl
Oligo Forward à 5µM	2,5 µl
Oligo Reverse à 5µM	2,5 µl
NEB 5X standard buffer One Taq	5 µl
dNTPs à 10mM each	0,5 µl
NEB OneTaq Hot-Start 5U/µl	0,13 µl
Total	25 µl

Tampon 5X	3,5 µl
Big Dye	1 µl
Primer à 5µM	0,6 µl
Produit de PCR purifié	1 à 2 µl
H20 QSP 20 µl	12,9 à 13,9 µl

Tableau S2

Sequence (5' to 3'): TTTCAACAAATCATAAAGATATTGG

Type: Primer

Length: 25

%GC: 24.0

Hairpin Tm: 35.4

Self Dimer Tm: None

Tm: 52.7

created by: primer3

Sequence (5' to 3'): TAAACTTCTGGGTGTCCGAAGAATCA

Type: Primer

Length: 26

%GC: 42.3

Hairpin Tm: 41.6

Self Dimer Tm: 17.2

Tm: 62.1

created by: primer3
