# Supplementary material for: What complete mitochondrial genomes tell us about the evolutionary history of the black soldier fly, Hermetia illucens
Source: BMC Ecol Evol. 2022 Jun 1;22:72. doi: 10.1186/s12862-022-02025-6 (PMC9158166; doi:10.1186/s12862-022-02025-6)
Supplement: Supplementary file 4 — Additional file 4. Annotated mitochondrial genome of Hermetia illucens. [file 12862_2022_2025_MOESM4_ESM.pdf]

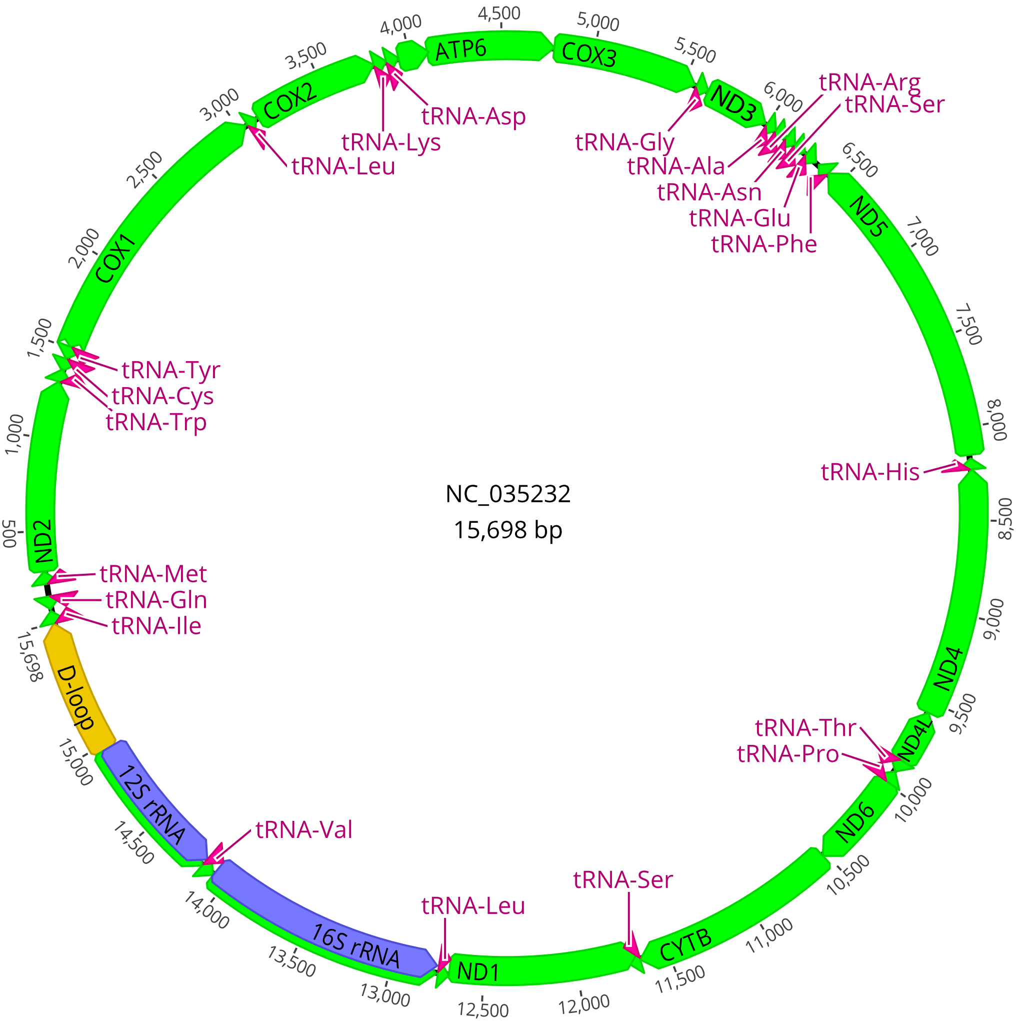


# Annotated mitochondrial genome of Hermetia illucens

The annotation of the genome was carried out using the MITOS tool.

Visualisation was carried out using the Geneious software.
