# Supplementary figures and images for: What complete mitochondrial genomes tell us about the evolutionary history of the black soldier fly, Hermetia illucens
Source: BMC Ecol Evol. 2022 Jun 1;22:72. doi: 10.1186/s12862-022-02025-6 (PMC9158166; doi:10.1186/s12862-022-02025-6)

Tree scale: 0.01

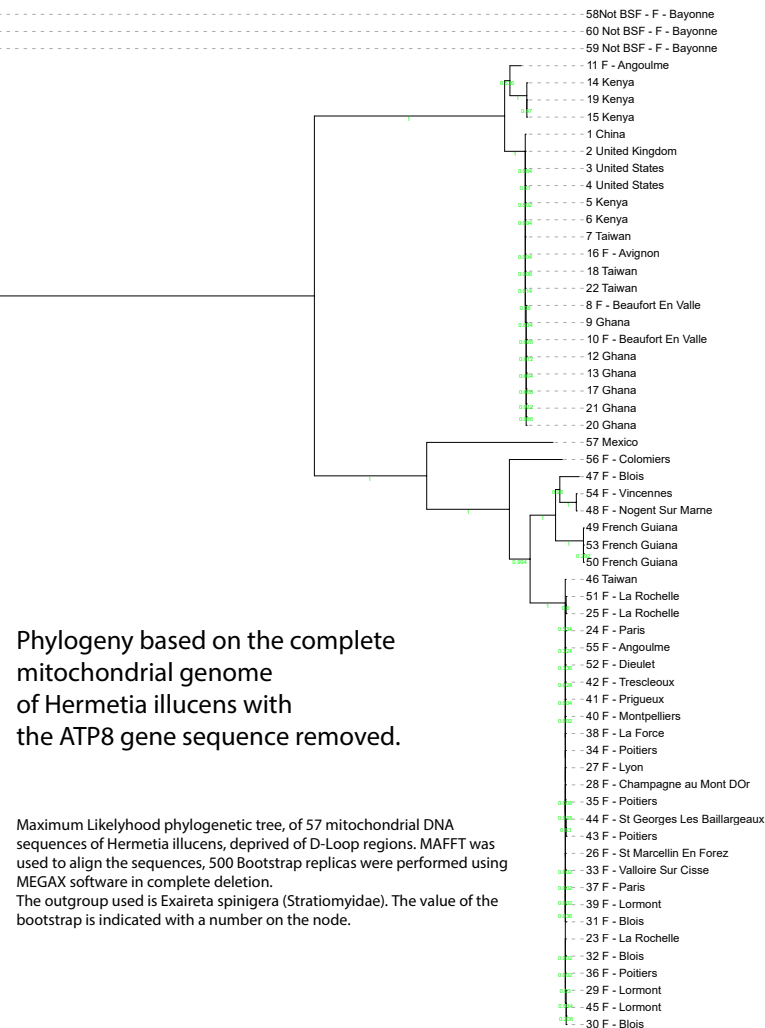

Supplement: Supplementary file 6 — Additional file 6. Phylogeny based on the complete mitochondrial genome of Hermetia illucens with the ATP8 gene sequence removed. [file 12862_2022_2025_MOESM6_ESM.pdf]

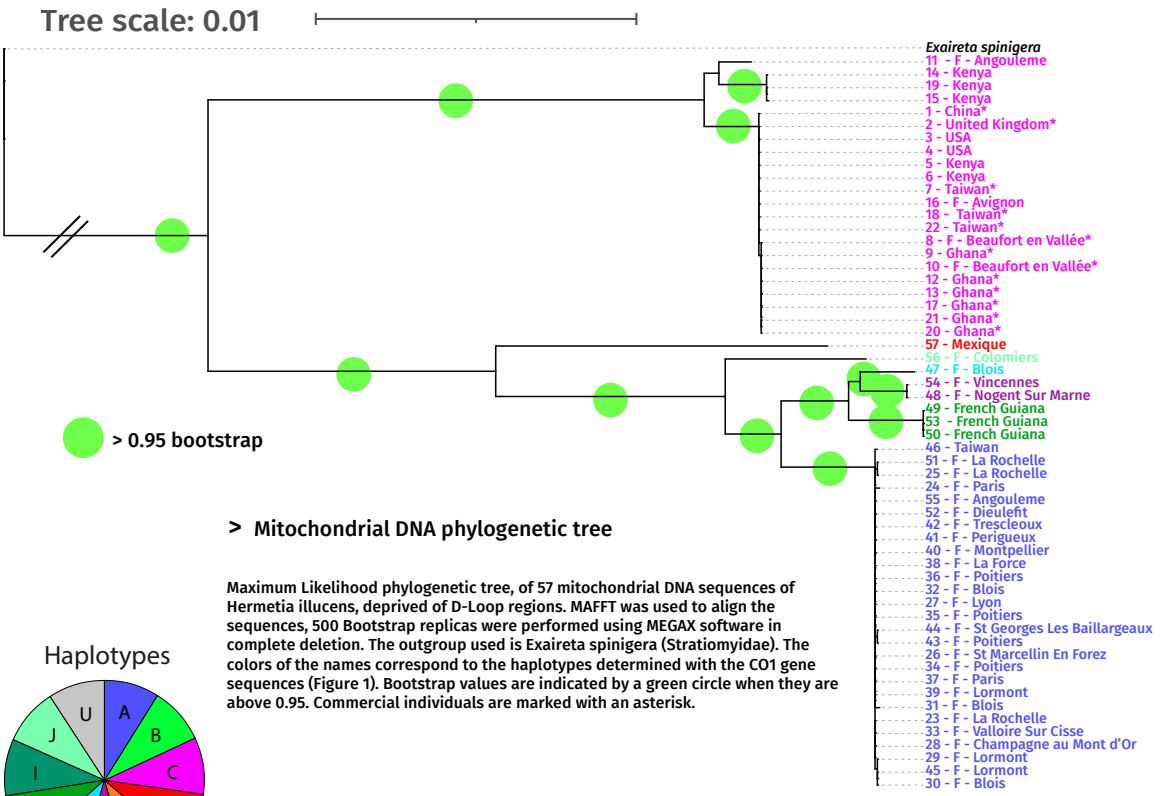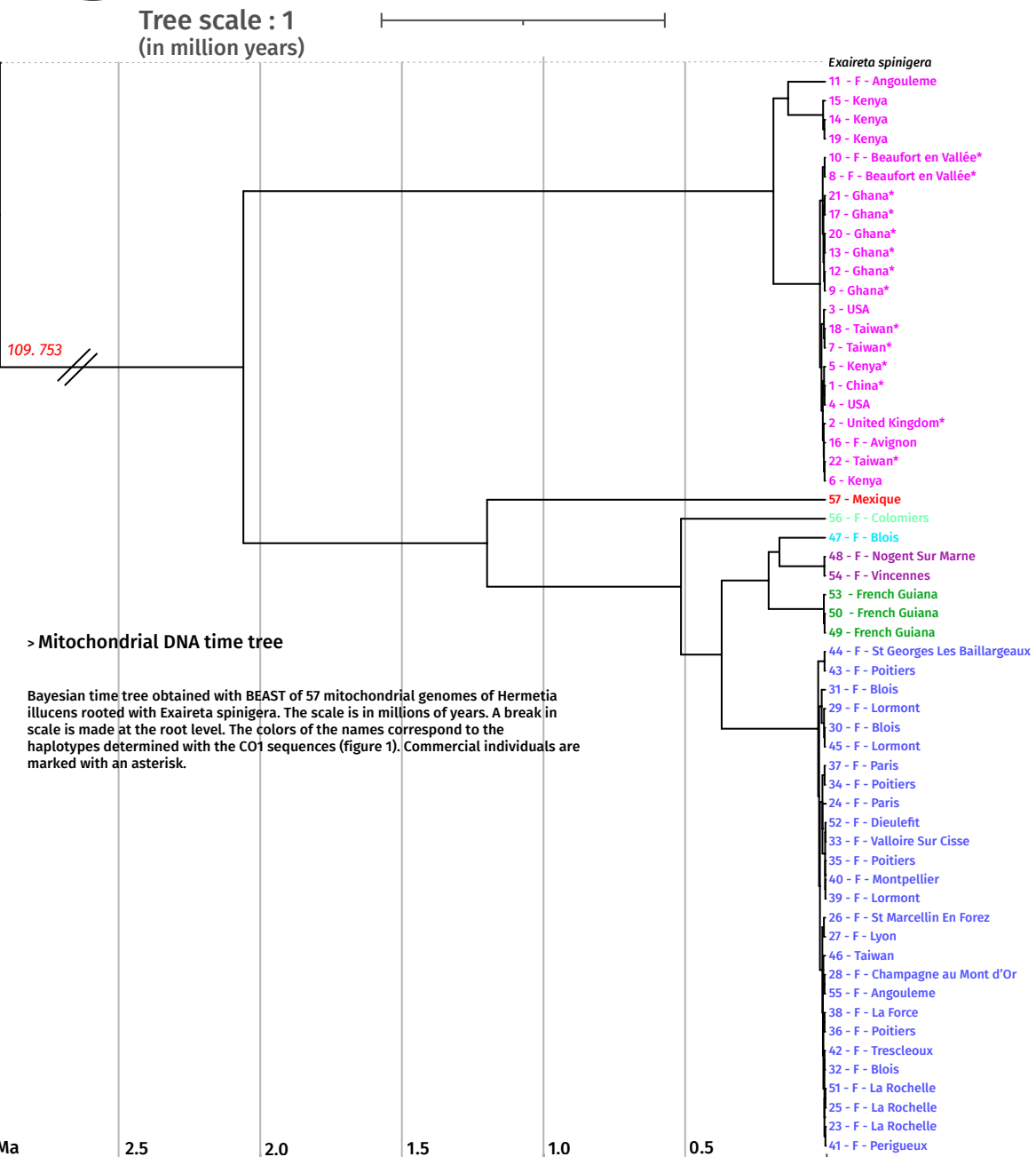

Supplement: Supplementary file 7 — Additional file 7. Mitochondrial DNA phylogenetic tree and mitochondrial DNA time tree. [file 12862_2022_2025_MOESM7_ESM.pdf]
