# Supplementary material for: What complete mitochondrial genomes tell us about the evolutionary history of the black soldier fly, Hermetia illucens
Source: BMC Ecol Evol. 2022 Jun 1;22:72. doi: 10.1186/s12862-022-02025-6 (PMC9158166; doi:10.1186/s12862-022-02025-6)
Supplement: Supplementary file 3 — Additional file 3. Phylogenetic tree of the CO1 gene. [file 12862_2022_2025_MOESM3_ESM.pdf]

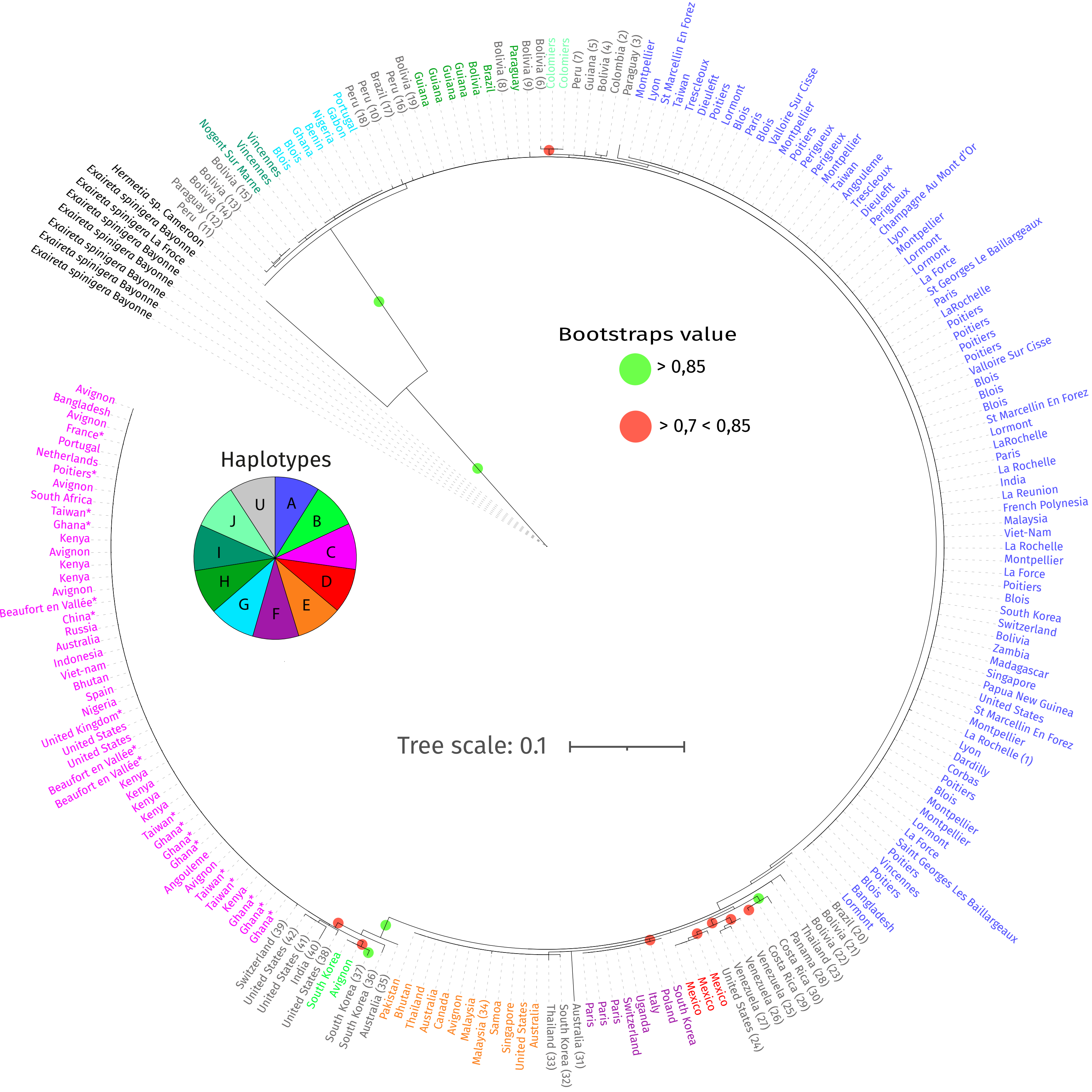

Phylogenetic tree of the CO1 gene.

This tree was made by keeping only one sequence per haplotype except for France.

The visualization is done using ITOL. An asterisk is placed for individuals from breeding.

The statistical support was estimated with 500 Bootstraps with MegaX software using the Tamura 92 G+I model.
